# Emission Rates of Volatile Organic Compounds from Humans

**DOI:** 10.1021/acs.est.1c08764

**Published:** 2022-04-07

**Authors:** Nijing Wang, Lisa Ernle, Gabriel Bekö, Pawel Wargocki, Jonathan Williams

**Affiliations:** † Atmospheric Chemistry Department, Max Planck Institute for Chemistry, Hahn-Meitner-Weg 1, 55128 Mainz, Germany; ‡ International Centre for Indoor Environment and Energy, Department of Environmental and Resource Engineering, Technical University of Denmark, Nils Koppels Alle 402, 2800 Lyngby, Denmark; § Climate & Atmosphere Research Centre, The Cyprus Institute, 1645 Nicosia, Cyprus

**Keywords:** ozone, clothing, temperature, relative humidity, breath, skin

## Abstract

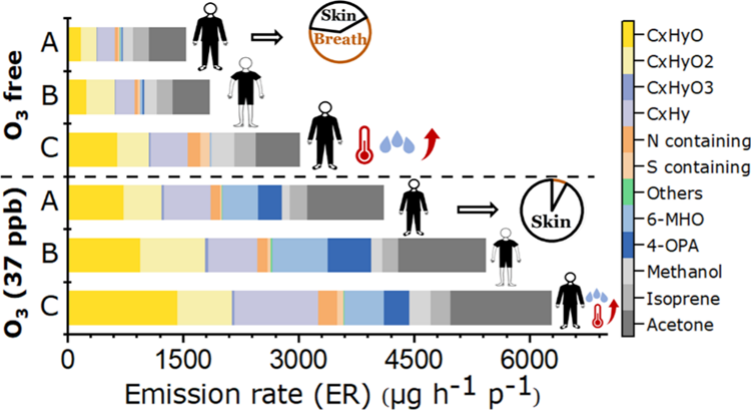

Human-emitted volatile organic compounds (VOCs) are mainly from breath and the skin. In this study, we continuously measured VOCs in a stainless-steel environmentally controlled climate chamber (22.5 m^3^, air change rate at 3.2 h^–1^) occupied by four seated human volunteers using proton transfer reaction time-of-flight mass spectrometry and gas chromatography mass spectrometry. Experiments with human whole body, breath-only, and dermal-only emissions were performed under ozone-free and ozone-present conditions. In addition, the effect of temperature, relative humidity, clothing type, and age was investigated for whole-body emissions. Without ozone, the whole-body total emission rate (ER) was 2180 ± 620 μg h^–1^ per person (p^–1^), dominated by exhaled chemicals. The ERs of oxygenated VOCs were positively correlated with the enthalpy of the air. Under ozone-present conditions (∼37 ppb), the whole-body total ER doubled, with the increase mainly driven by VOCs resulting from skin surface lipids/ozone reactions, which increased with relative humidity. Long clothing (more covered skin) was found to reduce the total ERs but enhanced certain chemicals related to the clothing. The ERs of VOCs derived from this study provide a valuable data set of human emissions under various conditions and can be used in models to better predict indoor air quality, especially for highly occupied environments.

## Abstract 2

Human chemical emission rates can be significantly affected by ozone and vary with temperature, relative humidity, and clothing coverage.

## 1 Introduction

Human beings are a potent mobile source of volatile organic compounds (VOCs) in the indoor environment. Several hundred VOCs are known to be emitted by people to their surrounding air via exhalation and dermal emissions.^1^ Although building materials, decorations, furniture, and consumer products have been also reported as important indoor VOC sources,^2−7^ the role of human beings as an indoor VOC source will likely become more important in the future due to regulatory measures to decrease emissions from indoor furnishings and building materials, coupled with reduced ventilation rates in modern energy-efficient buildings. Oxidants present in indoor air (e.g., ozone or hydroxyl radicals) can produce VOCs on the surface of human beings (including clothing, hair, and skin), as well as in the gas phase.^8−10^ Such surface generated compounds can continue to be produced for several days even after the occupants leave an indoor environment due to the transfer of skin lipids to other indoor surfaces.^11^ Some of these species have adverse effects on human health.^12^ For example, exposure to 4-oxopentanal (4-OPA), one of the major skin lipids ozonolysis products, can cause irritation and allergic responses^13^ and induce oxidative stress and inflammation in lung cells.^14^ Therefore, it is important to understand the speciation and the emission rates (ERs) of VOCs released from human beings and the effect of oxidants under various typical indoor conditions.

Human VOC ERs have been determined from measurements in several real-world indoor environments: a university classroom,^15^ a cinema,^16^ a gallery room in a museum,^17^ a university athletic center,^18^ a test house,^19^ and laboratory offices.^20^ The main VOC species measured (e.g., methanol, ethanol, monoterpenes, and siloxanes) showed large variations due to previous alcohol or food consumption and the use of personal care products. A few studies have also determined separate breath and dermal ERs of VOCs using environmentally controlled chambers.^21−24^ The effect of ozone on human VOC emissions has been intensively studied in simulated indoor environments both with soiled clothing^25−29^ and with human occupancy.^28,30,31^ However, a comprehensive chemical characterization of human ERs, including whole-body, breath-only, and dermal-only emissions, in a controlled environment with varying temperature, relative humidity (RH), ozone level, and clothing coverage is lacking. Such data are essential for the chemical environment in occupied indoor spaces to be modeled.

This study is a part of the Indoor Chemical Human Emissions and Reactivity (ICHEAR) project,^32^ which used online VOC measuring techniques to monitor the variation of VOCs emitted from human beings in a climate chamber under controlled conditions. The total ERs together with their species contributions for whole body, breath, and skin were derived under ozone-free and ozone-present conditions. In addition, we investigated the effect of varying temperature and RH, skin coverage with clothing, and age of human beings.

## 2 Methods

### 2.1 Chamber Experiments

Experiments were performed in two identical stainless-steel chambers under environmentally controlled conditions [temperature, RH, and air change rate (ACR)]. A detailed description of the experimental setup and procedures was given by Bekö et al.^32^ Experiments with duration of half-day and full-day were carried out. For half-day experiments, four volunteers wearing identical freshly laundered “long” clothing (pants, long sleeve shirts, and calf socks) or “short” clothing (shorts, t-shirts, and ankle socks) entered the chamber in the morning and stayed for about 3 h. The chamber was prepared either without or with ozone (∼100 ppb target in empty chamber) before the volunteers entered the chamber. During full-day experiments, the volunteers exited the chamber for a 15 min lunch break after the morning session (always with ozone absent). Ten minutes after they re-entered, ozone was added into the supply air at a constant rate (∼100 ppb target in empty chamber), reaching a steady-state concentration of 35–40 ppb in the occupied chamber. The volunteers stayed for around 2.5 h during the afternoon ozone-present condition. Separating breath and dermal emissions was achieved by having volunteers breathing through breathing masks connected by tubing to an identical twin chamber. The last 15 min before the volunteers exited the chamber were selected to represent the steady-state condition. In total, five different groups of four volunteers [3 groups of young adults (A1, A2, and A3), one group of teenagers (T4), and one group of seniors (S5)] participated in the experiments. Volunteers were asked not to use additional personal care products besides the provided paraben-, perfume-, and colorant-free products over the experimental period. Detailed information about the experimental conditions is listed in Table S1.

### 2.2 VOC Measurements

A proton transfer reaction time-of-flight mass spectrometer (8000, IONICON Analytik) was used to continuously measure VOCs in the chamber’s exhaust air (drift pressure 2.2 mbar, drift temperature 60 °C, E/N 137 Td). Proton transfer reaction time-of-flight mass spectrometry (PTR-ToF-MS) sampled the sub-flow of ∼100 mL min^–1^ via fluorinated ethylene propylene tubing (i.d. = 3.18 mm) from the main chamber exhaust line (flow rate 7 L min^–1^, length 5 m, i.d. = 12.7 mm). With protonated water (H_3_O^+^) as the primary ions, VOCs having proton affinity higher than water (697 kJ/mol) undergo proton-transfer reactions and are detected on their protonated mass to charge ratio (*m*/*z*) without significant fragmentation.^33^ The mass resolution was around 4000 at mass 96 amu. The measurement time resolution was 20 s and the mass range was up to 500 amu. Measured ions were attributed to chemical formulas based on the exact *m*/*z*, followed by the assignment of specific chemical species. As the mass becomes larger, the probability of the existence of isomeric compounds also increases. Therefore, chemical formulas having multiple isomeric compounds were only attributed to specific compounds if that compound had been previously reported as being related to human body emissions or human-involved ozone-initiated chemistry in the literature. Quantification of measured species including limit of detection (LOD) (see, Table S2) can be found in the Supporting Information.

To compensate for the inability of PTR-ToF-MS to quantify isoprene and propanal in this study (due to interferences at the corresponding masses), the mixing ratios of those two species were taken from a custom-made fast gas chromatograph–mass spectrometer (fast-GC) with LOD <25 ppt and total uncertainty <10%, which sampled the substream from the same main inlet as PTR-ToF-MS. The instrument uses liquid nitrogen for its internal three-step cryogenic preconcentration unit. Sampling time was 30 s and the time resolution of the fast-GC was 3 min running under the selected ion monitoring mode. A full calibration was performed every day. Details of the instrument operation were described elsewhere.^34^ The interferences stem from the fact that C_5_H_8_H^+^ (*m*/*z* 69.070) could be affected by contributions from fragments of long chain aldehydes^35^ and propanal cannot be separated from acetone by PTR-MS with H_3_O^+^ as the primary ions.

### 2.3 Other Measurements

CO_2_ was continuously monitored by a cavity ring-down spectrometer (Picarro G2401; Picarro Inc.) sampling the sub-flow from the same main inlet as the PTR-ToF-MS and the fast-GC. Ozone added into the supply air was generated by a Jelight 600 UV ozone generator (Jelight Company Inc.). The ozone level inside the chamber or supply air was monitored by a 2B Technologies model 205 ozone monitor (2B Technologies). Detailed information on those instruments can be found in Bekö et al.^32^

### 2.4 ER Calculation

The ER (μg h^–1^ p^–1^) of a VOC species *i* was calculated according to eq 1 by using the concentrations measured during the steady-state period, when the source of VOC was in equilibrium with the losses. As the human body (inhalation and skin surface) can also act as a sink of VOCs and indoor oxidants (ozone and OH radicals) can both produce and consume VOCs, the ERs derived in this study represent net emissions from human occupancy.
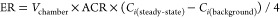
1*V*_chamber_ is the chamber volume (22.5 m^3^). ACR is the air change rate (3.2 h^–1^), which was determined from the decay of the CO_2_ concentration after the volunteers left the chamber, as well as by active tracer gas method using Freon 134a as tracer gas.^32^*C*_*i*(steady-state)_ represents the mean concentration of species *i* (μg m^–3^) over the steady-state period. *C*_*i*(background)_ is the mean background concentration of species *i* in the empty chamber before the volunteers entered the chamber for each experiment in the morning (15 min). Four is the number of volunteers in the chamber. In total, 179 species were identified and the full list of species can be found in Table S3 in the Supporting Information.

## 3 Results and Discussion

### 3.1 Time Series of Major VOCs Emitted by Humans

**Figure 1:**
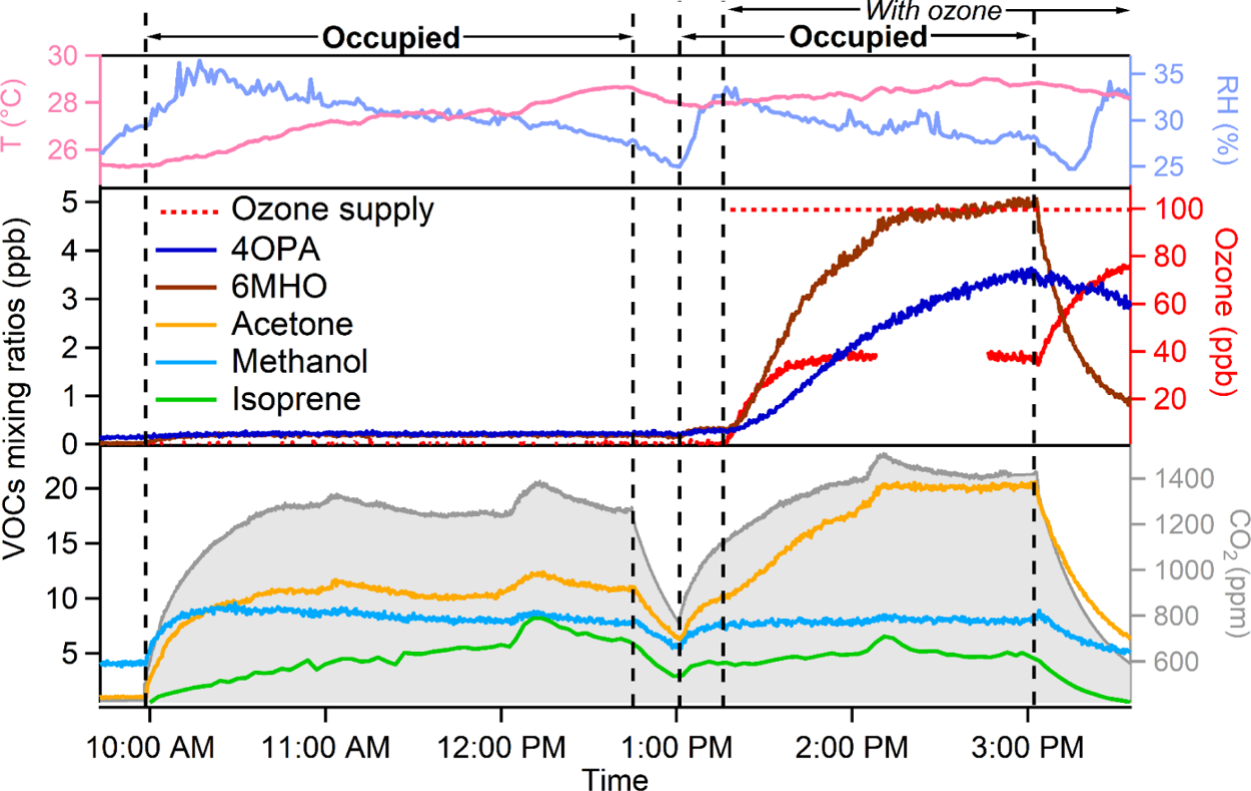
Time series of major VOCs and CO_2_ related to human emissions along with ozone, temperature (*T*), and RH in the chamber (data from Exp. 10).

Figure 1 illustrates an example of a typical experiment of whole-body emissions including several important marker compounds from breath and ozonolysis of skin surface lipids. Human exhaled endogenous compounds including acetone, isoprene, and methanol^36^ immediately increased along with CO_2_ when volunteers entered the chamber in the morning. This phenomenon was also observed when the volunteers re-entered the chamber in the early afternoon before ozone generation. The time required to reach relatively stable levels varied among compounds, which resulted from possible uptake by humans and surfaces, depending on compound solubility (detailed discussion in the Supporting Information). After ozone was introduced into the chamber, the known squalene ozonolysis products 6-methyl-5-hepten-2-one (6-MHO), acetone, and 4-OPA^31,37^ showed significant increases. The steady-state ozone level was ∼35 ppb in the afternoon, indicating a large ozone loss (around two-thirds) due to reaction with surfaces of the human occupants. After the volunteers had left the chamber, all major trace gases except for 4-OPA decreased rapidly. The simultaneous increase of CO_2_ and most of the VOC species for a short period in the middle of the morning and afternoon sessions were due to instructed coordinated movements by the volunteers (standing up and stretching), which increased their metabolic rate, breathing frequency, and possibly altered the airflow patterns inside the chamber.

### 3.2 Whole-Body, Breath, and Dermal ERs

**Figure 2:**
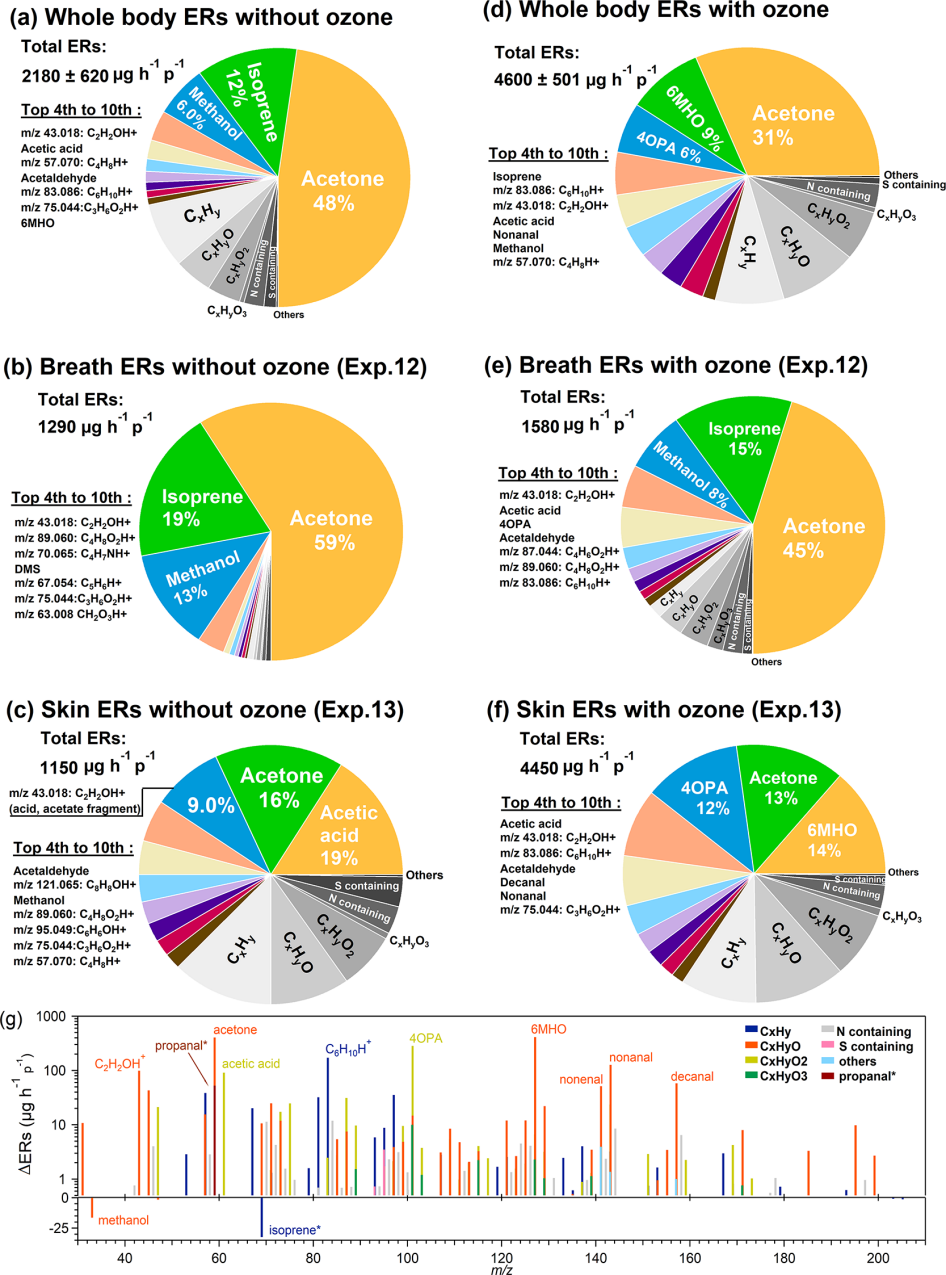
Under ozone-free conditions, total ERs and fractional contributions from top 10 species and other species for (a) whole-body emissions (mean values of Exp. 1, 6, 10, and 21; 21 was the replicate of 6), (b) breath emissions (Exp. 12), and (c) dermal emissions (Exp. 13). Corresponding whole-body, breath, and dermal emissions under the ozone-present condition, respectively, are shown to the right (panels d–f). The top three contributing species are labeled together with the percentage. Top 4th to 10th contributing species are listed for each condition. Mean absolute changes in ERs (panel g) with the presence of ozone (ΔERs) are shown for all measured species (averaged from Exp. 1, 6, 10, and 21). The 10 species having the most increases and 2 species having the most significant decrease are labeled. Propanal was measured by fast-GC.

Benchmark experiments (Exp. 1, 6, 21, and 10) for three different groups of volunteers (A1, A2, and A3) were included to derive the mean whole-body VOC ERs. The chemical composition and the steady-state ERs of most species were similar among these benchmarks except for acetone (Figure S1 in the Supporting Information). Acetone has been previously shown to be the major endogenous compound in breath and can vary from person to person and even with time for the same person due to differences in human metabolism.^1,38^ Without ozone, the total whole-body ER was 2180 ± 620 μg h^–1^ p^–1^ (Figure 2a). Top three contributors, acetone, isoprene, and methanol, accounted for 66% of the total whole-body ER. These compounds were also ranked as the top three species contributing to the breath-only ERs (Figure 2b), accounting for 91% of the total ER (1290 μg h^–1^ p^–1^). Only acetone was ranked among the three most contributing species to the total dermal emissions (ER of acetone: 180 μg h^–1^ p^–1^; Figure 2c). The top 10 species shown in Figure 2a–c accounted for 97, 63, and 78% of the total breath ER, skin ER, and whole-body ER, respectively. Thus, our findings further support the view that the chemical profile for dermal emissions is more diverse than for breath emissions.^1^ Besides the top 10 contributing species, the rest of the measured species was categorized into C_*x*_H_*y*_ (hydrocarbons), C_*x*_H_*y*_O_1–3_ (species containing one, two, or three oxygen atoms), species containing nitrogen, species containing sulfur, and others (rest of species). Dermal emissions were the main source of these species; the whole-body ER of the sum of these categories was 22% of the total ER (480 ± 54 μg h^–1^ p^–1^). It should be noted that the C_*x*_H_*y*_ subgroup is more likely to be alkyl fragments from other oxygenated VOCs rather than alkane/alkene, as the majority of them correlated well with the oxygenated VOCs. It has been reported in the literature that alcohols and aldehydes can fragment onto alkyl masses in PTR-MS.^35,39^

The total whole-body ER of group A3 was 1590 μg h^–1^ p^–1^ (Exp. 10), about 850 μg h^–1^ p^–1^ lower than the summed ER obtained from separate breath-only and dermal-only experiments performed with the same group of people, which might be partially explained by inhalation and dermal uptake.^40,41^ However, the dermal-only experiment (Exp. 13) was performed with short clothing, which could have enhanced the total ER (the effect of clothing is discussed in Section 3.4). In addition, day-to-day variation in emissions could further contribute to the discrepancy.

When ozone was present in the chamber, as shown in Figure 2f, the total skin-only ER increased from 1150 to 4450 μg h^–1^ p^–1^ with the presence of ozone. Organic acids were replaced by 6-MHO and 4-OPA among the top three contributors to the total skin ER. Other species among the top 10 most abundant emissions, including acetic acid, C_2_H_2_OH^+^ (acid fragment), C_6_H_10_H^+^ (general fragment of long-chain aldehydes), decanal, nonanal, and C_3_H_6_O_2_H^+^ (hydroxyacetone/propanoic acid), have all been reported as products of skin lipid ozonolysis.^31,42^ The effect of ozone on breath ERs was much smaller. Although ERs of endogenous breath compounds acetone, isoprene, and methanol slightly decreased, they remained as the top three contributors (Figure 2e). The decreased ER of isoprene is likely due to its reaction with oxidants (ozone and OH radicals), while the reaction of both oxidants with methanol and acetone is too slow to be significant. OH radicals generated from ozonolysis reactions can be an important loss route for isoprene compared to ozone due to its much faster reaction rate coefficient with OH radicals than with ozone (more details in the Supporting Information). Higher metabolic states in the afternoon may also be a reason for the changes in emissions of endogenous compounds. The steady-state CO_2_ was indeed always slightly higher after the lunch break in the afternoon.^32^ The breath-only ER increased in the afternoon by about 290 μg h^–1^ p^–1^, mainly due to oxygenated compounds. However, as the ozone was introduced into the chamber that only contained exhalation, those oxygenated compounds were more likely to be products of ozone reacting with the surfaces in the chamber soiled with residual human skin lipids from previous experiments. Therefore, only terminal compounds (without double bonds) from squalene ozonolysis, such as 4-OPA and C_4_H_6_O_2_H^+^ (1,4-butanedial), were among the top 10 contributing species (Figure 2e).

The mean total whole-body ER doubled with the presence of ozone (from 2180 to 4600 μg h^–1^ p^–1^). To better understand the speciated contribution to the increase in emissions induced by ozone, the change in ER for each species (ΔER = ER_ozone-present_ – ER_ozone-free_) was calculated for each benchmark experiment. Figure 2g shows the mean ΔERs of all species. More than 100 species and ions had an increased ER, while a limited number of species demonstrated a decreased ER (mainly methanol and isoprene) with ozone present in the chamber. Species with an increase in ER above 10 μg h^–1^ p^–1^ were mainly hydrocarbon fragments and oxygenated VOCs containing one or two oxygen atoms. Interestingly, the ERs of some nitrogen containing species were also elevated under ozone-present conditions. They were probably generated from reactions of emitted ammonia^43^ with ozonides produced from skin/ozone reactions.^44^ The VOC ERs did not correlate strongly with the ammonia ER (*r*^2^ ≤ 0.5 under ozone-free condition and *r*^2^ ≤ 0.4 under ozone-present condition across all experiments performed under moderate temperature). The top 10 elevated species listed in Figure 2g mostly agreed well with the top 10 species of skin-only emissions when ozone was present (Figure 2f), indicating the total ΔER of whole-body emissions was mainly contributed by those compounds produced from skin/ozone reactions.

### 3.3 Effect of Temperature and RH on ERs

The effect of indoor temperature and RH on human emissions was studied using the data obtained from one set of experiments with the same group of volunteers (A1). The steady-state temperature and RH varied from 29.3 to 32.6 °C and 32 to 62%, respectively.

**Figure 3:**
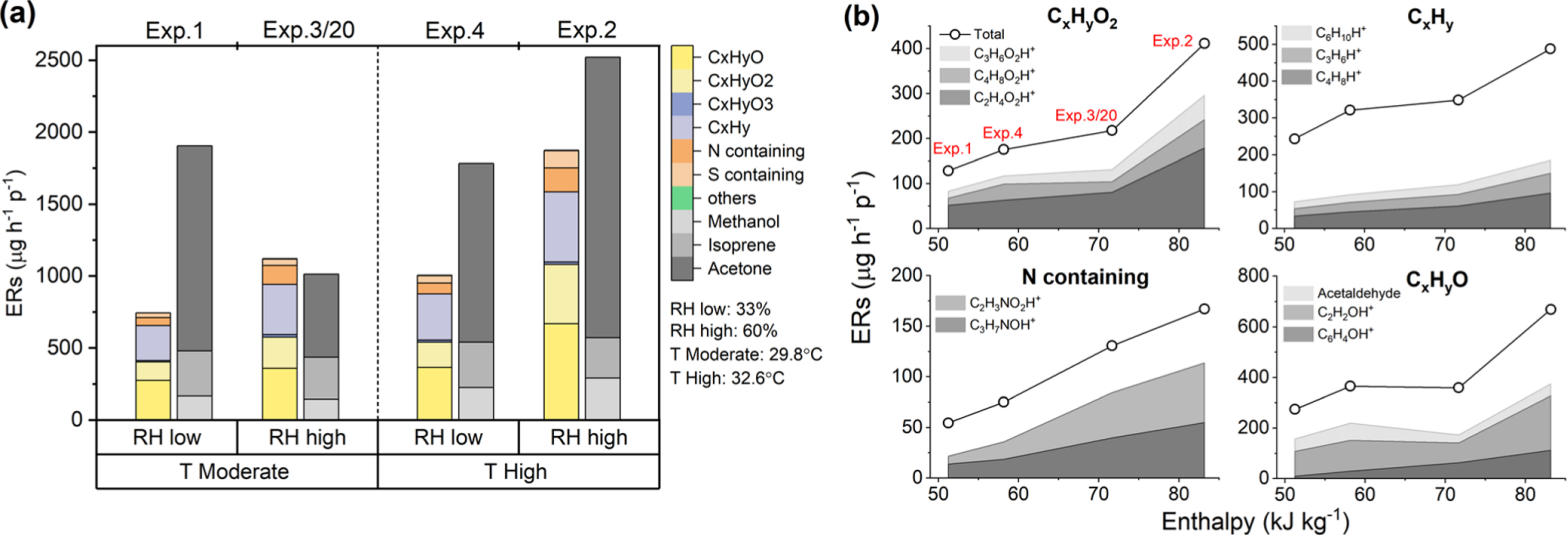
(a) Whole-body ERs of acetone, isoprene, and methanol and other subgroups under the ozone-free condition with different temperatures (*T*) and RH levels. (b) Whole-body ERs of the most contributing subgroups (empty circles and line) and the top contributing species to each subgroup (stacked area) under ozone-free conditions as a function of enthalpy. Exp. 20 is the replicate of Exp. 3 and the mean values of these two experiments are shown in the plots.

Under ozone-free condition, as shown in Figure 3a, the top three contributing species, acetone, isoprene, and methanol, did not show any clear dependency on either temperature or RH. At the same RH level, the sum of the remaining species showed a higher ER under the high temperature condition. It is known that a higher temperature can enhance the release of VOCs into the gas phase and less water moisture in the air can reduce the partitioning of water-soluble compounds into the aqueous phase. However, our results showed that with the same temperature, higher RH enhanced the ER of remaining species, indicating that the increase of temperature and RH altered the VOC ERs from humans. Based on the temperature and RH, we calculated the enthalpy (*H*) of the air for each experiment using the HumidAir psychrometric calculator.^45^ As the enthalpy increased, an increasing trend for the ER of the main contributing subgroups was observed as shown in Figure 3b. The top contributing species (ions) listed for each subgroup in Figure 3b were also reported as microbial volatiles emitted from skin microbes.^46^ Experiments done with another group of volunteers focusing on skin-only emissions showed that the total ER in Exp. 11 (temperature: 31 °C, RH: 70%, *H*: 83 kJ kg^–1^) was ∼400 μg h^–1^ p^–1^ higher than the total ER in Exp. 13 (temperature: 29 °C, RH: 28%, *H*: 47 kJ kg^–1^) and the increase was mainly caused by the same dominating species from each subgroup shown in Figure 3b (see Table S4). In addition, some of the volunteers may have begun to sweat under conditions with elevated temperature and RH. Carboxylic acids are one of the major components of human sweat.^47,48^ VOCs can further be generated by skin microbiota, contributing to body odor.^49^ The three most abundant species in the C_*x*_H_*y*_O_2_ group with the chemical formula of C_*n*_H_2*n*_O_2_ are likely to be acetic acid, propanoic acid, and butyric acid, all of which have been identified in human sweat samples.^47^ However, because sweating was not monitored in our study, we are not able to draw conclusions about the role sweating may have played in the observed human VOC emissions. In conclusion, elevated temperature and humidity, as reflected by the enthalpy, can possibly enhance human dermal emissions, which might be related to skin microbe activities as well as changes in human body metabolism. More experiments under different temperatures and RH values are needed to better understand the underlying mechanisms.

When ozone was introduced to the chamber, as shown in Figure S2 in the Supporting Information, the largest total ΔER (3270 μg h^–1^ p^–1^) was observed under the condition with high temperature and RH (Exp. 2, temperature: 32 °C, RH: 63%). Under moderate RH (32% for Exp. 4 and 30% for Exp. 1) and comparable temperatures (31.8 °C for Exp. 4 and 30.3 °C for Exp. 1), the total ER and the fractional contributions from subgroups were lower. Experiments probing skin-only emissions also resulted in higher total ΔER in Exp. 11 with higher RH (temperature: 31 °C, RH: 70%) compared to Exp. 13 (temperature: 30 °C, RH: 28%) (Figure S2). The top 10 species with highest ΔERs under the ozone-present condition were nearly identical for these two experiments and their ERs were elevated under high RH (right panel, Table 1). The overall yield (ppb VOC per ppb ozone) of those species increased as well (see Table S5, Supporting Information). In the whole-body experiments, ΔERs of 6-MHO, 4-OPA, nonanal, decanal, and C_6_H_10_H^+^ were elevated under high RH (left panel, Table 1). The results are consistent with a recent laboratory study showing that during squalene ozonolysis, the total mass concentration of gas-phase VOCs increased with the increase in water vapor.^50^ The authors suggested that the Criegee intermediates generated from primary ozonides generate more carbonyls and less secondary ozonides with increased water vapor.^50^ Thus, our results confirmed that increased RH indoors can enhance the generation of gas-phase products from skin lipid ozonolysis.

**Table 1:** Top 10 Species with Highest ΔERs under the Ozone-Present Condition Relative to the Corresponding Ozone-Free Condition at Different Temperatures (*T*) and RH

whole-body emission (long clothing)				skin-only emission (short clothing)			
		ΔERs (μg p–^1^ h–^1^)				ΔERs (μg p–^1^ h–^1^)	
top ten species for Exp. 4 **(rankings for Exp. 2)**		*T*: 32 °C RH: 30% (Exp. 4^a^)	*T*: 32 °C RH: 63% (Exp. 2^b^)	top ten species for Exp. 13 **(rankings for Exp. 11)**		*T*: 30 °C RH: 28% (Exp. 13)	*T*: 31 °C RH: 70% (Exp. 11^c^)
1	acetone **(1)**	820	740	1	6-MHO **(1)**	590	740
2	6-MHO **(2)**	370	500	2	4-OPA **(2)**	530	690
3	4-OPA **(3)**	230	320	3	acetone **(3)**	420	550
4	C_6_H_10_H^+^**(5)**	180	280	4	acetic acid **(4)**	190	510
5	nonanal **(4)**	140	310	5	C_2_H_2_OH^+^**(5)**	180	400
6	C_2_H_2_OH^+^**(7)**	120	130	6	C_6_H_10_H^+^**(6)**	150	260
7	acetic acid **(8)**	120	120	7	decanal **(7)**	87	220
8	propanal **(11)**	67	53	8	nonanal **(8)**	76	180
9	acetaldehyde **(12)**	62	47	9	1,4-butanedial **(13)**	57	78
10	decanal **(6)**	61	140	10	nonenal **(9)**	56	130

a Exp. 4 was chosen for comparison to represent the moderate temperature condition because the steady-state temperature in Exp. 4 (31.8 °C) was closer to the temperature in Exp. 2 (32.3 °C) compared to the temperature in Exp. 1 (30.3 °C).

b In Exp. 2 (whole-body emission), nonenal (100 μg p^–1^ h^–1^), and C_7_H_12_H^+^ (59 μg p^–1^ h^–1^) were ranked 9th and 10th.

c In Exp. 11 (skin-only emission), acetaldehyde (100 μg p^–1^ h^–1^) was ranked 10th.

### 3.4 Effect of Clean Clothing on ERs

**Figure 4:**
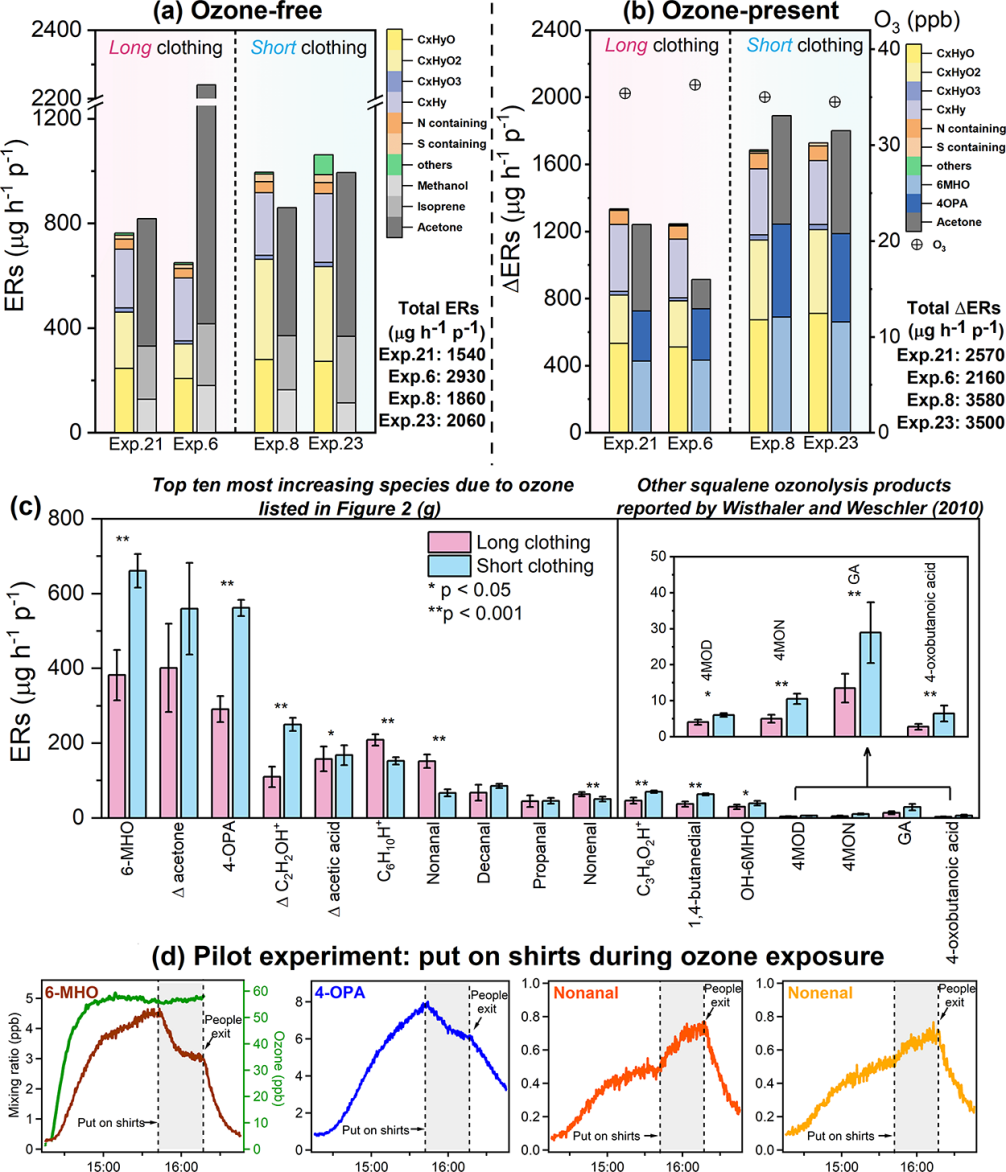
(a) Fractional contributions to whole-body ERs under the ozone-free condition for long and short clothing, (b) fractional contributions to the absolute change of whole-body ERs (ΔERs) under the ozone-present condition for long and short clothing as well as steady-state ozone mixing ratios for each experiment (Exp. 6 and 21, Exp. 8 and 23 are replicates, respectively), (c) mean ERs of top 10 most increasing species in the presence of ozone (listed in Figure 2g) and of other squalene ozonolysis products reported by Wisthaler and Weschler^31^ under the ozone-present condition with long clothing (*N* = 12) and short clothing (*N* = 4), where Δ represents the ΔER was used for that species (*N* = 8 for long clothing and *N* = 2 for short clothing) and (d) time series of selected trace gas mixing ratios from a pilot experiment with clean shirts putting on during ozone exposure.

Experiments performed with volunteer group A2 focused on the effect of clothing under ozone-free and ozone-present conditions. As shown in Figure 4a, when the chamber was free of ozone, the ERs of main endogenous exhaled species (acetone, isoprene, and methanol) showed no clear difference between long and short clothing. However, the total ER of the remaining species increased from 710 μg h^–1^ p^–1^ wearing long clean clothing (mean of Exp. 6 and its replicate Exp. 21) to 1030 μg h^–1^ p^–1^ wearing short clean clothing (mean of Exp. 8 and its replicate Exp. 23). The increase was mainly driven by species in the group of C_*x*_H_*y*_O_2_, where C_2_H_4_O_2_ (acetic acid) accounted for 30% of the increase. As shown in Figure 2c, acetic acid had the highest ER during the skin-only experiment without ozone. Previous research has shown that clean clothing can absorb chemicals in the air and reduce dermal uptake.^51^ Clean clothing can thus also act as a barrier for chemicals released from the skin into the air, leading to lower ERs of dermally emitted compounds with long clothing.

Under ozone-present conditions, as shown in Figure 4b, the total emissions increased considerably and the mean ΔER with short clothing was 1170 μg h^–1^ p^–1^ higher than the mean ΔER with long clothing. The top three species, 6-MHO, acetone, and 4-OPA, are known squalene ozonolysis products, and they accounted for 52% of the total ER increase. The remaining fraction was attributable mainly to C_*x*_H_*y*_O_2_ and C_*x*_H_*y*_O. However, the ozone consumption was nearly the same for the experiments with different clothing coverage, reflecting the fact that ozone reactions also occur on the surfaces of clothing and they are mass transport limited.^27^

For the same clothing type, the fractional contributions to the total ERs as well as the ERs for the top 10 most increasing species (listed in Figure 2g) were similar for the experiments in which the steady-state ozone level was established before the volunteers entered the chamber (“from start”) and the experiments in which ozone dosing started 10 min after the volunteers re-entered the chamber (“from SS”) (Figure S3). Therefore, for each clothing type, we included all experiments performed at a moderate temperature and RH (across all five volunteer groups: *N* = 12 experiments with long clothing, *N* = 4 experiments with short clothing) to calculate the mean ERs of each top 10 most increasing species as well as of other squalene ozonolysis products reported by Wisthaler and Weschler.^31^ For acetone, acetic acid, and C_2_H_2_OH^+^, due to their unneglectable contribution observed during ozone-free condition as shown in Figure 2, we used ΔERs from experiments of “from SS” instead of the ERs. Most of the species known as skin lipid ozonolysis products had significantly higher ERs (*p* < 0.05, details of statistical analysis are shown in the Supporting Information) when wearing short clothing compared to wearing long clothing (Figure 4c). Although acetone is one of the terminal products generated from squalene ozonolysis,^37^ the mean of ΔER of acetone was not significantly higher when wearing short clothing rather than long clothing (*p* = 0.08). This may be due to variations in exhaled acetone levels.

Nonanal, nonenal, as well as the general chain aldehydes fragment ion C_6_H_10_H^+^ showed the opposite trend, where significantly higher ERs were observed when long clothing was worn. This was confirmed in a separate pilot experiment where two male adults (wearing shorts only) were sitting for 1.5 h inside the chamber with ozone present and then put on clean shirts (stored in sealed plastic bags until wearing). As shown in Figure 4d, 6-MHO and 4-OPA immediately dropped, while nonanal and nonenal increased. This indicates that clothing can compete with skin lipids for reacting with ozone and is responsible for the majority of the nonanal and nonenal yield under the ozone-present condition. This finding agrees with previous studies where long chain aldehydes (nonanal in particular) were measured when exposing laundered cotton fabric to ozone.^25,26^ Nonanal and nonenal are products of ozone reacting with oleic acid and linoleic acid.^52^ These unsaturated fatty acids only contribute a minor fraction to human skin lipids; decanal is the most dominant compound from the ozonolysis of unsaturated fatty acids in skin lipids.^10^ It is therefore likely that the higher ERs of the two C9 species is due to the use of natural oils during the textile processing and to the natural occurrence of unsaturated fatty acids in cotton as well as in detergent residue.^53−55^ In conclusion, when exposing human beings to ozone, more clothing can reduce chemicals generated from skin/ozone reactions, but it can increase the presence of other specific chemicals originating from the clothing.^56^

### 3.5 ERs of Volunteers with Different Ages

Experiments with volunteers of different ages under the same chamber conditions and clothing type were selected to investigate the effect of age. Four benchmark experiments and two morning-only experiments with ozone were performed with young adults (*N* = 6, with three groups of volunteers, average age: 25.1). Two benchmark experiments and one morning-only experiment with ozone were performed with teenagers (average age: 13.8) and seniors (average age: 70.5) (*N* = 3 for both groups). Under the ozone-free condition, different age groups showed similar fractional contributions of emitted compounds to whole-body ERs (Figure S4 in the Supporting Information). No significant difference was identified for the total ERs or the ER for each subgroup among different age groups. Among the top three contributing species, only methanol had a significantly higher ER among teenagers compared to seniors (*p* = 0.018; Figure S4c). Methanol is an endogenous compound observed in exhalation^36^ and has been reported to have an inverse correlation with the body mass index (BMI).^57^ The senior group had the highest mean BMI of 25.6 (BMI of young adults: 21.6; teenagers: 19.5), which is in line with previous findings. When ozone was present, the total ERs nearly doubled, and there were no significant differences between the three groups of volunteers with different ages. However, the relatively small difference in the ER of 6-MHO between seniors (310 ± 33 μg h^–1^ p^–1^) and young adults (430 ± 58 μg h^–1^ p^–1^) and in the ER of 4-OPA between seniors (260 ± 18 μg h^–1^ p^–1^) and teenagers (310 ± 11 μg h^–1^ p^–1^) reached statistical significance (Figure S4c). The abundance of skin lipids has been found to decrease with the increase in age,^58^ which may result in less lipid ozonolysis among the seniors.^59^

### 3.6 Comparison of ERs with the Literature

In our study, exogenous sources of human emissions were minimized by the controlled use of personal care products and clothing, and certain dietary restrictions.^32^ Meanwhile, some VOCs have relatively low volatility (“sticky compounds”); the concentration of those species may not have reached steady state. Therefore, the total whole-body ERs reported in our study are the lower limit of the human ERs. Tang et al.^15^ reported total VOC ER measured by PTR-MS in a classroom of 6250 μg h^–1^ p^–1^. Cyclic volatile methylsiloxanes (cVMS) commonly present in personal care products constituted the largest contribution (45%).^15^ By subtracting the ER of cVMS, the total ER in the classroom would be 3450 μg h^–1^ p^–1^, which is higher than the whole-body ER under the ozone-free condition in the present study (2170 ± 980 μg h^–1^ p^–1^). Although the exact ozone level in the classroom was not reported, ozone loss along with the increase in the mixing ratio of squalene ozonolysis products (6-MHO and 4-OPA) was observed when the classroom was occupied.^15^ Thus, the higher ER obtained in the classroom was likely caused by products of skin lipid ozonolysis as well as other VOCs emitted from personal care products.

**Figure 5:**
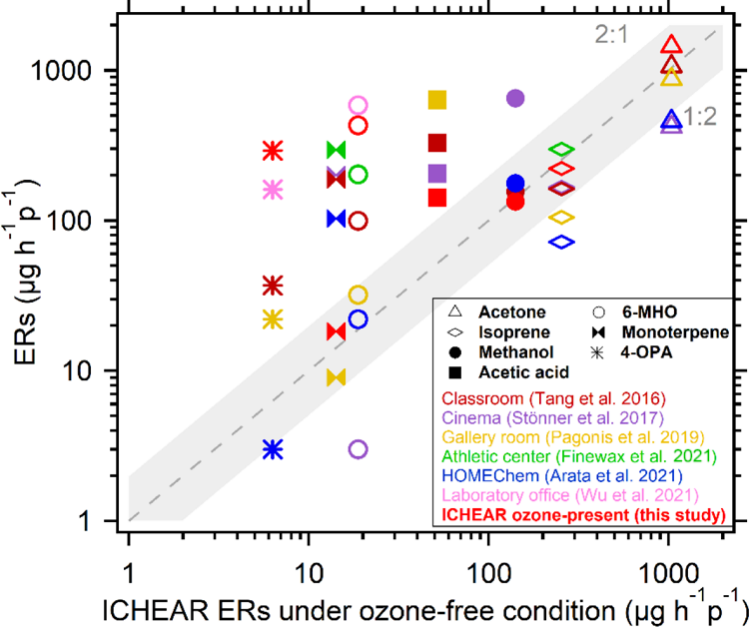
Comparison of selected VOC ERs in this study (ozone-free condition on the horizontal axis) with other studies and this study’s ozone-present condition (vertical axis).

Several studies have reported human ERs measured with PTR-MS in various indoor environments.^15−20^ A comparison of these ERs for selected VOCs with our results is shown in Figure 5. The ERs of 6-MHO and 4-OPA in other indoor environments were mostly within the range of the ERs under ozone-free and ozone-present conditions in our study. This is understandable given the relatively high steady-state ozone concentration in our chamber (∼37 ppb) compared to other indoor environments.^60^ In an occupied cinema, 4-OPA was not detected and the ER of 6-MHO was lower than that in our study without ozone.^16^ The authors attributed the low ER to limited indoor ozone due to low outside ozone in the inner city.^16^ Large surface areas present in the cinema (e.g., seats and carpet) might also contribute to the loss of ozone. Because the experiment in the morning was performed without ozone, the ERs of 6-MHO and 4-OPA were expected to be very low. The observed elevated ERs at the absence of ozone in our study compared to other studies was probably due to exposure of the volunteers to ozone prior to entering the chamber.

The ERs of the endogenous breath compounds acetone, isoprene, and methanol were very close to those reported in other studies. One exception is the much higher ER of methanol in the cinema, where the methanol emission (which peaked in early screenings) was suggested to be more likely an exogenous compound due to fruit or fruit juice intake at breakfast. Although acetone can also be generated via squalene ozonolysis, the dominant source of acetone in the other studies is likely to be human breath because the ozone levels were much lower than in our study. Acetic acid can be emitted from exhalation and skin^1^ or generated by reactions of ozone with skin oils.^42^ The ER reported in other studies is generally higher than our ER, presumably due to the presence of additional sources to those of the human beings alone. A study done in a museum gallery room, which had the highest ER of acetic acid among the studies reviewed, demonstrated that alcohol consumption before the opening event may have contributed to acetic acid levels,^17^ as ethanol metabolism produces acetic acid that can subsequently be detected in human breath.^62^

Due to the restricted use of personal care products in our study, the ERs of monoterpenes were much lower than the ERs reported in other studies, but comparable to the ER in a museum gallery room with low occupant density. Little use of personal care products by the visitors and their declining ER over the day was suggested (the event in the gallery was held in late afternoon).^17^ Only few studies have characterized the ERs of breath-associated and skin-associated VOCs in an occupied sealed chamber and the ozone level was kept as low as possible.^23,61,63^ We compare the reported ERs of selected VOCs with our data from the breath-only and dermal-only experiments under the ozone-free condition. ERs in our study are in general agreement with previous reported values. Details are shown in the Supporting Information (Tables S6 and S7).

### 3.7 Limitations and Implications

The uncertainty for the ER of species that were not calibrated using gas standards can be up to 50%. It can be especially high when fragmentation occurs using H_3_O^+^ as the reagent ions in PTR-MS. The total uncertainty of whole-body ER under the ozone-free condition will be lower than that under the ozone-present condition because the fractional contribution of standard-calibrated species was higher under the ozone-free condition compared to the ozone-present condition. Better quantification of the major contributing species, especially under the ozone condition, would help to lower the uncertainty of the human ERs. In addition, limited replicated experiments could introduce extra uncertainty of the ERs.

With the specially designed chamber experiments and real-time VOC measurements, this study was able to characterize human bioeffluents and the effect of temperature, RH, clothing, and volunteers’ age under ozone-free and ozone-present conditions on human ERs. The obtained ERs of VOCs can help to improve existing indoor air quality models and assessments of humans as chemical emission sources in various indoor environments.

## Supporting Information Available

Mixing ratio calculation of compounds measured with PTR-ToF-MS; statistical analysis; varying time to reach steady-state level for endogenous breath compounds; gas-phase chemistry case study: isoprene and 6-MHO; breath and dermal ERs reported in the literature; experiments reported in this study; LOD and total uncertainty of the calibrated species for PTR-ToF-MS; whole-body ERs of VOCs under ozone-free and ozone-present conditions; ERs of the most contributing subgroups during skin-only experiments under different temperature and relative humidity conditions as well as the enthalpy of the air; yield of top 10 species having the most increase under ozone-present condition for experiments of skin-only emissions with moderate and high relative humidity; human breath ERs of selected species in the literature; human dermal ERs of selected species in the literature; human ERs under the ozone-free condition and ΔERs under ozone-present condition; fractional contributions to ΔERs for whole-body and skin-only emissions under different temperature and relative humidity under ozone-present condition; fractional contributions to total ERs as well as the ERs of the top ten species (excluding acetone) for two types of experiments, wearing long clothing and short clothing ; fractional contributions to the human whole-body ERs of teenagers, young adults, and seniors under ozone-free and ozone-present conditions, and ERs of selected species for different age groups.; and associated references (PDF)
