# Supplementary material for: Emission Rates of Volatile Organic Compounds from Humans
Source: Environ Sci Technol. 2022 Apr 7;56(8):4838–48. doi: 10.1021/acs.est.1c08764 (PMC9022422; doi:10.1021/acs.est.1c08764)
Supplement: Supplementary file 1 — es1c08764_si_001.pdf [file es1c08764_si_001.pdf]

Supporting Information

Emission Rates of Volatile Organic Compounds from Humans

Nijing Wang[1*], Lisa Ernle[1], Gabriel Bekö[2], Pawel Wargocki[2], Jonathan Williams[1, 3]

[1]Atmospheric chemistry department, Max Planck Institute for Chemistry, Hahn-Meitner-Weg 1, 55128, Mainz, Germany

[2]International Centre for Indoor Environment and Energy, Department of Environmental and Resource Engineering, Technical University of Denmark, Nils Koppels Alle 402, 2800, Lyngby, Denmark

[3]Climate & Atmosphere Research Centre, The Cyprus Institute, 1645, Nicosia, Cyprus

*corresponding author: nijing.wang@mpic.de

Pages: S1 – S17

Tables: S1, S2, S3, S4, S5, S6, S7

Figures: S1, S2, S3, S4

• Mixing ratio calculation of compounds measured with PTR-ToF-MS

A full calibration including the humidity dependence (RH ranging from 15% to 80%) was performed at the start, in the middle and at the end of the campaign by using a standard gas mixture containing methanol, acetonitrile acetaldehyde, acetone, dimethyl sulfide (DMS), isoprene, methyl vinyl ketone (MVK), methacrolein, methyl ethyl ketone (MEK), benzene, toluene, xylene, 1,3,5-Trimethylbenzene and α-pinene (Apel-Riemer Environmental Inc.). Aromatic compounds were found to have the largest sensitivity decrease as the humidity increases (up to 20%). The limit of detection of calibrated species ranged from 8 ppt for 1,3,5-trimethylbenzene to 171 ppt for methanol, with the uncertainty ≤11% (Table S2). PTRwid was used to perform the data processing where a default mass-dependent transmission efficiency was applied (Holzinger, 2015). The default correction parameterization was derived based on the method reported by Cappellin et al. (2012), where the same type of instrument (PTR-ToF-MS 8000, Ionicon Analytik) was used under similar operating conditions to those of our study. To verify the accuracy of the default transmission efficiency correction, we back calculated the experimental reaction rate coefficient of VOC reacting with H_3O^+ based on the known VOC mixing ratio of the gas standard and the VOC mixing ratio derived from PTRwid. For non-fragmenting species (e.g. acetonitrile, acetaldehyde, acetone, methyl ethyl ketone, benzene and 1,3,5-trimethylbenzene), the relative difference between the experimental reaction rate and the theoretic reaction rate (reported by Cappellin et al. (2012)) was in the range of 1% to 10%, indicating that the default transmission curve agreed well with the actual situation. For VOC species without gas standard calibration, the mixing ratios were calculated based on a theoretical method by using a constant proton transfer reaction rate coefficient of 2×10^{-9} cm^3 $molecule^{-1}$ s^{-1} with the uncertainty of ~ 50% (Sekimoto et al., 2017; Zhao and Zhang, 2004). In addition, major fragmentation of aldehydes losing one water molecule was considered for those species (marked with * in Table S3) to better quantify their levels. Those fragments were tested in the lab or reported as the fragment using PTR-MS (reference details are listed in the footnote of Table S3).

• Statistical analysis

One-way analysis of variance (ANOVA) was applied to identify the statistical differences between long and short clothing as well as age groups for each selected VOC. The Tukey test was used for

the mean comparisons. A p-value <0.05 represents significant difference. The analysis was performed with OriginLab (version: 2019b).

- **Varying time to reach steady-state level for endogenous breath compounds**

After the volunteers entered the chamber with no ozone present, the rate at which mixing ratios increased differed between endogenous breath compounds. Methanol was found to approach equilibrium in the shortest time (~20 minutes), followed by acetone (~30 minutes), CO_2 (~45 minutes) and isoprene (~60 minutes). In order to identify whether the chamber surfaces contributed to this effect, we derived the decay rate for each compound from the data after the volunteers had left the chamber. This was done for one experiment (Exp. 20) without ozone. We found that the decay rate for each compound was similar (<8%) to the air change rate at 3.2 h^{-1}, indicating that the contribution of the chamber surfaces being a sink or source was negligible for these three compounds. Therefore, the main factor driving the time to steady-state for different compounds is likely to be deposition in the human respiratory system. It has been reported that the mixing ratio of a compound in exhaled air is nearly in linear relationship with the mixing ratio of that compound in inhaled air, and generally the more water-soluble the compound is, the larger uptake by the upper airways is expected (Ager et al., 2020; Spanel et al., 2013). Methanol has the highest Henry's law constant at body temperature, followed by acetone, CO_2 and isoprene (Kramer et al., 2016). Therefore, as the uptake by inhalation is most significant for methanol, it reaches steady-state in the shortest time.

- **Gas-phase chemistry case study: isoprene and 6-MHO**

In this study, ozone was the main oxidant driving ozonolysis reactions involving unsaturated compounds. Many VOCs are not only products but also precursors of other VOCs (Wisthaler and Weschler, 2010). Hydroxyl radicals can be formed during ozonolysis (Finlayson-Pitts and Pitts Jr, 1999). When ozone was present in the chamber, 6-MHO and isoprene were the most abundant compounds containing C=C double bond. Assuming that 6-MHO and isoprene are the only precursors for OH radical generation, we estimated the OH radical concentration for Exp.10 (shown in Figure 1) to be 2.5×10^{-5} ppb, using the method reported by Weschler and Shields (1996). The total OH radical loss frequency at steady state in Exp.10 was taken from Wang et al. (2021). Using the rate coefficient of 6-MHO reacting with ozone and OH (3.9×10^{-16} cm^3

molecules^{-1} s^{-1} and 1.6×10^{-10} cm^3 molecules^{-1} s^{-1}, respectively (Smith et al., 1996)), and the rate coefficient of isoprene reacting with ozone and OH (1.3×10^{-17} cm^3 molecules^{-1} s^{-1} and 1.0×10^{-10} cm^3 molecules^{-1} s^{-1}, respectively (IUPAC)), the loss rate of 6-MHO via ozone and OH was 1.29 h^{-1} and 0.34 h^{-1} respectively, and the loss rate of isoprene via ozone and OH was 0.04 h^{-1} and 0.22h^{-1} respectively. Therefore, the loss of isoprene by ozone oxidation was much smaller compared to OH oxidation under the chamber conditions, and both loss rates were substantially smaller than the air change rate in the chamber (3.2 h^{-1}). 6-MHO was consumed by ozone at a higher rate compared to OH, and the loss rate due to ozone reactions was of the same magnitude as due to ACR. The results indicate that 6-MHO plays a more important role than isoprene in the presence of ozone in occupied indoor environments.

- **Breath and dermal emission rates (ERs) reported in the literature**

ERs of common exhalation and skin emitted VOCs measured in this study were compared with those in the literature, as shown in Table S6 and Table S7. Because ozone levels in most studies were kept as low as possible, we only list the ERs we obtained under ozone-free conditions. The breath ER of isoprene was higher than the mean values reported by Sun et al. (2017) and the ER of monoterpenes was lower. They are however within the range of values measured in 117 volunteers. The ER of acetone reported by Sun et al. (2017) showed little variation among individuals and is much lower compared to the ER of acetone in our study. The authors claimed that semi-quantification of acetone might be a reason, as the calibration linear fit was not good enough. Other VOCs were not detected in our study, which may be due to lack of repetitive experiments among different volunteer groups. For dermal emissions (Table S6), our results generally agree better with the results from Mochalski et al. (2014) except for 6-MHO and hexanal. Exposure to ozone before the experiments might be the reason for the much higher 6-MHO ER even under ozone-free condition. The generally lower ERs of VOCs reported by Zou et al. (2020) was probably due to showering directly before the experiment. The ERs of long chain aldehydes were found in general to be lower than the values reported in the two other studies. Bias from the quantification using the assumed constant proton transfer reaction rate coefficient instead of gas standards can also be a contributory factor.

Table S1. Experiments reported in this study: modified from Table 1 in Bekö et al. (2020)

Subject group	Exp. No.	Steady-state temperature (afternoon) (°C)	Steady-state relative humidity (afternoon) (%)	Steady-state ozone (afternoon) (ppb)	Clothing L (long) S (short)	Note
A1 (young adults)	1	29.3 (30.3)	33 (32)	<1 (34.1)	L	whole-body
	2	32.6 (32.3)	62 (63)	<1 (38.8)	L	
	3	29.4	62	<1	L	
	4	32.5 (31.8)	32 (30)	<1 (33.6)	L	
	20	30.9	56	<1	L	replicate of Exp. 3
A2 (young adults)	6	26.1 (27.5)	20 (18)	<1 (36.3)	L	whole-body
	7	26	27	34.8	L	
	8	27.2 (28.4)	24 (23)	<1 (35)	S	
	9	26.5	20	36.9	S	
	21	26.2 (27.7)	24 (21)	<1 (35.4)	L	replicate of Exp. 6
	22	27.1	20	39.6	L	replicate of Exp. 7
	23	27.7 (28.9)	22 (22)	<1 (34.5)	S	replicate of Exp. 8
	24	27.1	19	36.3	S	replicate of Exp. 9
A3 (young adults)	10	28.6 (28.8)	28 (28)	<1 (37.6)	L	whole-body
	11	31.1 (30.8)	70 (70)	<1 (38)	S	dermal only
	12	32.3 (32.2)	60 (57)	<1 (96.5)	S	breath only
	13	28.9 (29.6)	28 (28)	<1 (36.3)	S	dermal only
T4 (teenagers)	18	27.5 (28.4)	35 (35)	<1 (37.3)	L	whole-body
	19	28.5	34	44.2	L	
	26	29.3 (30)	33 (35)	<1 (40.6)	L	replicate of Exp. 18
S5 (seniors)	16	28.9 (29.7)	28 (25)	<1 (37.7)	L	whole-body
	17	28.2	24	43.1	L	
	25	28.3 (29)	25 (25)	<1 (41.4)	L	replicate of Exp. 16

Table S2. Limits of detection and total uncertainty of the calibrated species for PTR-ToF-MS

m/z (H^+)	Compounds	LOD (3σ) ppt	Total uncertainty %
33.034	Methanol	171	11
42.033	Acetonitrile	26	7
45.033	Acetaldehyde	130	6
59.049	Acetone	84	6
63.026	Dimethyl Sulfide (DMS)	14	6
71.049	Methyl vinyl ketone/Methacrolein	35	5
73.065	Methyl ethyl ketone	30	6
79.054	Benzene	11	6
93.070	Toluene	11	6
107.086	Xylene	10	6
121.101	1,3,5-Trimethylbenzene	8	7
137.132	Monoterpenes	11	6

Table S3. Whole-body emission rates (μg h^{-1} p^{-1}) of VOCs (mean values of Exp. 1, 6, 10 and 21) under ozone-free and ozone-present conditions.

m/z	Formula	Ozone-free		Ozone-present		Potential compound
		Mean	STD	Mean	STD	
31.018	CH_2OH^+	3.8	1.7	15	3.8	formaldehyde
33.034	CH_4OH^+	140	41	130	40	methanol[#]
42.033	$C_2H_3NH^+$	2.1	1.0	2.7	1.3	acetonitrile[#]
43.018	$C_2H_2OH^+$	82	27	180	24	general fragment
43.054	$C_3H_6H^+$	19	1.4	20	1.3	
45.033	$C_2H_4OH^+$	29	14	72	15	acetaldehyde[#]
46.029	CH_3NOH^+	2.2	0.9	5.9	1.2	formamide/formaldoxime
47.013	$CH_2O_2H^+$	N	N	23	10	formic acid
47.049	$C_2H_6OH^+$	2.2	0.6	1.4	0.1	ethanol
49.011	CH_4SH^+	0.5	0.2	0.6	0.1	methanethiol
53.039	$C_4H_4H^+$	1.2	0.2	4.0	0.4	
57.033	$C_3H_4OH^+$	4.5	0.4	20	2.1	acrolein
57.070	$C_4H_8H^+$	34	1.1	72	4.9	general fragment
58.040	$CH_3N_3H^+$	0.8	0.3	3.6	0.7	
59.049	$C_3H_6OH^+$	1040	700	1440	620	acetone[#]
61.028	$C_2H_4O_2H^+$	52	5.6	140	16	acetic acid
63.008	$CH_2O_3H^+$	4.0	0.6	4.4	0.6	
63.026	$C_2H_6SH^+$	5.6	0.6	5.7	0.7	dimethyl sulfide [#]
67.054	$C_5H_6H^+$	11	0.7	31	2.1	
69.033	$C_4H_4OH^+$	2.7	0.7	13	1.9	furan
71.013	$C_3H_2O_2H^+$	0.7	0.2	2.0	0.3	propiolic acid
71.049	$C_4H_6OH^+$	9.9	4.3	35	6.1	methyl vinyl ketone[#], methacrolein[#]
71.086	$C_5H_{10}H^+$	6.7	1.6	7.8	2.0	
72.044	$C_3H_5NOH^+$	0.9	0.4	5.2	0.8	acrylamide
73.028	$C_3H_4O_2H^+$	2.3	0.5	20	1.7	acrylic acid, methyl glyoxal
73.065	$C_4H_8OH^+$	7.4	2.3	19	1.8	methyl ethyl ketone[#]
74.024	$C_2H_3NO_2H^+$	6.8	1.8	7.0	2.1	nitroethene
74.060	$C_3H_7NOH^+$	2.6	1.7	6.7	6.6	propanamide
75.044	$C_3H_6O_2H^+$	19	2.9	44	4.3	hydroxyacetone, propanoic acid
76.039	$C_2H_5NO_2H^+$	0.8	0.2	1.8	0.1	glycine (amino acid)
77.022	$C_2H_4O_3H^+$/ $CH_4N_2SH^+$	0.7	0.2	1.2	0.2	glycolic acid /thiourea
79.054	$C_6H_6H^+$	2.1	1.1	3.3	0.5	benzene[#]
81.045	$C_4H_4N_2H^+$	0.7	0.2	1.3	0.3	
81.070	$C_6H_8H^+$	9.3	0.8	41	1.9	
83.013	$C_4H_2O_2H^+$	N	N	2.7	0.2	
83.086	$C_6H_{10}H^+$	23	4.1	190	14	general fragment

84.044	$C_4H_5NOH^+$	N	N	3.6	0.3	
84.081	$C_5H_9NH^+$	1.7	0.3	14	0.8	
85.028	$C_4H_4O_2H^+$	2.2	0.5	4.9	0.9	
85.065	$C_5H_8OH^+$	2.9	0.6	8.3	0.8	C5 unsaturated carbonyl
85.101	$C_6H_{12}H^+$	2.8	0.3	3.1	0.4	
86.060	$C_4H_7NOH^+$	N	N	1.1	0.2	
87.044	$C_4H_6O_2H^+$	6.6	1.2	38	3.6	1,4-butanedial
87.080	$C_5H_{10}OH^+$	2.2	0.3	9.8	1.7	C5 aliphatic carbonyl (e.g. pentanal)
88.076	$C_4H_9NOH^+$	0.4	0.0	1.0	0.2	
89.023	$C_3H_4O_3H^+$	2.2	0.8	3.7	0.7	
89.060	$C_4H_8O_2H^+$	17	11	27	16	C4 acid or ester
91.058	$C_4H_{10}SH^+$	8.0	5.2	8.2	2.9	1-(methylthio)-propane
93.033	$C_6H_4OH^+$	7.8	3.8	8.4	4.1	
93.070	$C_7H_8H^+$	2.2	0.5	8.1	1.1	toluene#
94.065	$C_6H_7NH^+$	0.4	0.1	1.1	0.1	
95.016	$C_2H_6O_2SH^+$	10	3.5	14	4.0	dimethyl sulfone
95.049	$C_6H_6OH^+$	5.1	4.0	6.5	4.0	phenol
95.086	$C_7H_{10}H^+$	7.6	0.8	16	1.4	
96.044	$C_5H_5NOH^+$	0.6	0.2	0.7	0.3	
96.081	$C_6H_9NH^+$	0.8	0.1	3.1	0.4	
97.028	$C_5H_4O_2H^+$	1.5	0.4	3.6	0.7	
97.065	$C_6H_8OH^+$	1.8	0.3	5.7	0.4	
97.101	$C_7H_{12}H^+$	11	1.4	46	3.3	
98.071	$C_4H_7N_3H^+$	N	N	1.3	0.1	
98.096	$C_6H_{11}NH^+$	0.8	0.1	3.9	0.4	
99.045	$C_5H_6O_2H^+$	2.1	0.4	12	1.0	4-oxo-2-pentenal
99.080	$C_6H_{10}OH^+$	2.1	0.2	7.0	0.7	C6 unsaturated carbonyls
100.039	$C_4H_5NO_2H^+$	0.8	0.1	2.1	0.3	
100.076	$C_5H_9NOH^+$	0.5	0.0	0.9	0.3	
101.023	$C_4H_4O_3H^+$	N	N	11	3.8	
101.060	$C_5H_8O_2H^+$	8.9	1.6	290	35	4-oxopentanal (4-OPA)*
101.096	$C_6H_{12}OH^+$	1.4	0.2	16	3.7	C6 aliphatic carbonyl (e.g. hexanal)
103.039	$C_4H_6O_3H^+$	2.0	1.5	3.2	1.2	4-oxobuntanoic acid
103.075	$C_5H_{10}O_2H^+$	2.9	0.2	6.6	0.5	C5 acid or ester
105.037	$C_4H_8OSH^+$/ $C_3H_8N_2SH^+$	0.7	0.3	1.1	0.3	
105.070	$C_8H_8H^+$	1.8	0.5	1.9	0.4	styrene
107.049	$C_7H_6OH^+$	4.2	1.1	7.3	1.6	benzaldehyde
107.086	$C_8H_{10}H^+$	1.3	0.2	4.1	0.4	xylene#
109.028	$C_6H_4O_2H^+$	N	N	5.7	0.8	

109.065	$C_7H_8OH^+$	1.1	0.3	9.6	0.9	
111.044	$C_6H_6O_2H^+$	0.7	0.1	1.7	0.2	
111.081	$C_7H_{10}OH^+$	1.2	0.3	6.0	0.6	
111.117	$C_8H_{14}H^+$	6.9	1.0	22	2.4	
112.112	$C_7H_{13}NH^+$	0.5	0.1	2.0	0.2	
113.060	$C_6H_8O_2H^+$	1.1	0.2	2.9	0.2	
113.096	$C_7H_{12}OH^+$	0.9	0.2	3.0	0.5	
115.037	$C_5H_6O_3H^+$	N	N	2.8	0.3	
115.075	$C_6H_{10}O_2H^+$	1.8	0.4	5.9	0.8	
115.112	$C_7H_{14}OH^+$	0.6	0.1	3.8	0.5	C7 aliphatic carbonyls (e.g. heptanal)
117.091	$C_6H_{12}O_2H^+$	2.7	0.2	5.2	0.4	C6 acid or ester
119.086	$C_9H_{10}H^+$	1.7	0.3	3.4	0.3	
121.065	$C_8H_8OH^+$	6.6	5.7	19	7.7	
121.101	$C_9H_{12}H^+$	1.8	0.4	4.4	0.7	1,3,5-trimethylbenzene[#]
122.060	$C_7H_7NOH^+$	1.1	0.5	2.0	0.8	
123.044	$C_7H_6O_2H^+$	1.3	0.1	2.7	0.7	
123.080	$C_8H_{10}OH^+$	0.7	0.1	3.4	0.4	
124.112	$C_8H_{13}NH^+$	0.6	0.1	5.1	0.6	
125.035	$C_5H_4N_2O_2H^+$	N	N	1.2	0.2	
125.060	$C_7H_8O_2H^+$	0.7	0.2	2.5	0.3	
126.103	$C_6H_{11}N_3H^+$	0.3	0.1	3.5	0.2	
126.128	$C_8H_{15}NH^+$	0.4	0.0	4.5	0.3	
127.039	$C_6H_6O_3H^+$	N	N	2.6	0.3	
127.075	$C_7H_{10}O_2H^+$	1.2	0.4	7.7	1.1	
127.112	$C_8H_{14}OH^+$	19	6.7	430	50	6-methyl-5-hepten-2-one (6MHO)*
129.052	$C_6H_8O_3H^+$	0.8	0.1	1.8	0.3	
129.091	$C_7H_{12}O_2H^+$	1.3	0.3	4.3	0.7	
129.127	$C_8H_{16}OH^+$	7.9	1.3	30	3.1	C8 aliphatic carbonyls (e.g. octanal)
131.070	$C_6H_{10}O_3H^+$	0.7	0.3	1.0	0.2	
131.118	$C_6H_{14}N_2OH^+$	1.2	0.1	1.9	0.6	
133.101	$C_{10}H_{12}H^+$/ $C_5H_{12}N_2O_2H^+$	1.4	0.4	3.9	0.7	
135.081	$C_9H_{10}OH^+$	0.6	0.1	1.6	1.2	
135.117	$C_{10}H_{14}H^+$	2.3	0.3	2.6	0.4	
137.060	$C_8H_8O_2H^+$	1.1	0.6	1.8	0.6	
137.132	$C_{10}H_{16}H^+$	14	2.5	18	1.4	monoterpenes[#]
138.128	$C_9H_{15}NH^+$	0.8	0.1	1.8	0.1	
139.039	$C_7H_6O_3H^+$	1.4	0.8	2.3	1.4	
139.075	$C_8H_{10}O_2H^+$	2.2	1.8	3.5	1.7	
139.112	$C_9H_{14}OH^+$	1.6	0.1	5.1	0.3	
141.066	$C_6H_8N_2O_2H^+$	0.8	0.2	4.7	0.8	

141.127	$C_9H_{16}OH^+$	7.5	0.3	59	6.2	C9 unsaturated carbonyl (e.g. nonenal)*
143.032	$C_6H_6O_4H^+$	N	N	1.6	0.4	
143.086	$C_{11}H_{10}H^+$	1.2	0.3	4.2	1.0	
143.107	$C_8H_{14}O_2H^+$	3.3	1.3	27	3.9	1-hydroxy-6-methyl-5-hepten-2-one (OH-6MHO)*
143.143	$C_9H_{18}OH^+$	7.8	0.6	140	16	C9 aliphatic carbonyl (e.g. nonanal)*
144.150	$C_7H_{17}N_3H^+$	0.4	0.0	8.9	0.4	
145.122	$C_8H_{16}O_2H^+$	2.0	0.7	3.3	0.6	C8 aliphatic acid or ester
149.035	$C_7H_4N_2O_2H^+$	1.0	0.4	1.4	0.3	
149.096	$C_{10}H_{12}OH^+$	0.9	0.2	1.1	0.2	
149.133	$C_{11}H_{16}H^+$	2.1	0.3	2.1	0.3	
151.147	$C_{11}H_{18}H^+$	2.8	0.3	3.4	0.2	
153.070	$C_{12}H_8H^+$	0.6	0.0	2.1	0.3	
153.127	$C_{10}H_{16}OH^+$	0.8	0.2	1.8	0.3	
155.107	$C_9H_{14}O_2H^+$	1.1	0.2	4.3	0.7	4-methyl-4-octene-1,8-dial (4-MOD)*
155.143	$C_{10}H_{18}OH^+$	1.2	0.3	4.6	0.5	C10 unsaturated carbonyls
157.047	$C_6H_8N_2OSH^+$	N	N	1.4	0.4	
157.159	$C_{10}H_{20}OH^+$	8.0	1.8	66	4.1	C10 aliphatic carbonyls (e.g. decanal)*
158.165	$C_8H_{19}N_3H^+$	0.7	0.2	7.1	0.6	
159.137	$C_9H_{18}O_2H^+$	0.9	0.2	3.2	0.2	C9 aliphatic acid or ester
163.148	$C_{12}H_{18}H^+$	1.7	0.4	1.7	0.3	
165.163	$C_{12}H_{20}H^+$	2.7	0.2	2.8	0.4	
167.086	$C_{13}H_{10}H^+$	0.7	0.1	3.5	0.5	
169.122	$C_{10}H_{16}O_2H^+$	N	N	5.8	0.8	4-methyl-8-oxo-4-nonenal (4-MON)*
169.170	$C_{10}H_{20}N_2H^+$	0.7	0.2	1.4	0.5	
171.102	$C_9H_{14}O_3H^+$	1.0	0.1	1.6	0.1	
171.175	$C_{11}H_{22}OH^+$	2.0	0.6	10	0.7	C11 aliphatic carbonyls (e.g. undecanal)
173.154	$C_{10}H_{20}O_2H^+$	1.0	0.3	1.9	0.4	C10 aliphatic acid or ester
177.051	$C_{10}H_8O_3H^+$	0.9	0.2	1.3	0.3	
178.159	$C_{12}H_{19}NH^+$	0.6	0.2	1.6	0.4	
179.179	$C_{13}H_{22}H^+$	2.3	0.3	2.7	0.2	
185.190	$C_{12}H_{24}OH^+$	3.0	0.7	6.3	0.8	C12 aliphatic carbonyls (e.g. dodecanal)
191.179	$C_{14}H_{22}H^+$	1.6	0.2	1.7	0.2	
193.195	$C_{14}H_{24}H^+$	1.7	0.2	2.0	0.4	
195.174	$C_{13}H_{22}OH^+$	6.6	2.0	16	2.2	geranyl acetone (GA)*
197.201	$C_{12}H_{24}N_2H$+	1.2	0.3	2.2	0.3	
199.205	$C_{13}H_{26}OH^+$	8.2	2.2	11	2.3	C13 aliphatic carbonyls (e.g. tridecanal)
201.185	$C_{12}H_{24}O_2H^+$	1.4	0.3	1.5	0.5	C12 aliphatic acid or ester

203.179	$C_{15}H_{22}H^+$	2.8	0.3	2.1	0.3	
205.195	$C_{15}H_{24}H^+$	4.7	0.9	3.5	0.4	
207.186	$C_{13}H_{22}N_2H^+$	2.0	0.5	1.9	0.5	
211.148	$C_{16}H_{18}H^+$	1.0	0.1	1.8	0.3	
213.160	$C_{16}H_{20}H^+$	2.4	0.5	2.8	0.2	
217.191	$C_{16}H_{24}H^+$	2.9	0.2	2.8	0.2	
219.207	$C_{16}H_{26}H^+$	3.6	0.3	2.9	0.5	
221.201	$C_{14}H_{24}N_2H^+$	2.0	0.3	1.5	0.3	
231.210	$C_{17}H_{26}H^+$	4.1	0.4	3.6	0.6	
233.226	$C_{17}H_{28}H^+$	5.6	0.6	4.9	0.6	
245.226	$C_{18}H_{28}H^+$	5.4	0.6	5.2	0.9	
247.242	$C_{18}H_{30}H^+$	6.8	0.5	6.6	0.9	
249.257	$C_{18}H_{32}H^+$	3.5	0.4	3.1	0.6	
253.288	$C_{18}H_{36}H^+$	2.2	0.7	1.9	0.6	
257.248	$C_{16}H_{32}O_2H^+$	22	41	25	45	C16 aliphatic acid or ester
259.242	$C_{19}H_{30}H^+$	5.5	1.3	5.7	1.3	
261.257	$C_{19}H_{32}H^+$	5.4	1.0	5.6	1.0	
263.273	$C_{19}H_{34}H^+$	3.2	0.8	3.0	0.6	
273.257	$C_{20}H_{32}H^+$	3.2	0.5	3.3	0.7	
275.273	$C_{20}H_{34}H^+$	3.2	0.9	3.3	1.0	
277.289	$C_{20}H_{36}H^+$	1.8	0.6	1.9	0.5	
289.288	$C_{21}H_{36}H^+$	1.4	0.1	1.5	0.4	
303.304	$C_{22}H_{38}H^+$	N	N	0.4	0.0	
371.101	$C_{10}H_{30}O_5Si_5H^+$	0.9	0.4	0.6	0.0	D5*
445.120	$C_{12}H_{36}O_6Si_6H^+$	0.8	0.4	0.7	0.2	D6*
	propanal (GC)	3.5	3.1	59	11	
	isoprene (GC)	270	59	250	55	

¶: Only species having yield larger than 0.001 are shown.

N: no increase due to human occupancy.

# VOCs calibrated with the gas standard.

* The main fragmentation ion is also considered in the calculation (tested in the lab; **reported by Wisthaler and Weschler (2010)**; *reported by Zhou et al. (2014)*):

4-OPA (m/z 83.049), 6-MHO (m/z 109.101*), nonenal (m/z 123.117)*, **OH-6MHO (m/z 125.096)**, nonanal (m/z 125.132), **4-MOD (m/z 137.096)**, decanal (m/z 139.148), **4-MON (m/z 151.112), GA (m/z 177.164)**, D5 (m/z 355.070) and D6 (m/z 429.090).

Table S4. Emission rates (µg p^{-1} h^{-1}) of the most contributing subgroups during skin-only experiments under different temperature (T) and relative humidity (RH) conditions as well as the enthalpy (H) of the air. Species refer to the top three contributing species to each subgroup as mentioned in the text in section 3.3. The same volunteers participated in Exp. 11 and Exp. 13 and they wore short clothing.

**Subgroup**	**Exp.13** T: 29°C RH: 28% H: 47 kJ kg^{-1}	**Exp. 11** T: 31°C RH: 70% H: 83 kJ kg^{-1}	**Species**	**Exp.13** T: 29°C RH: 28% H: 47 kJ kg^{-1}	**Exp. 11** T: 31°C RH: 70% H: 83 kJ kg^{-1}
$CxHyO_2$	320	490	$C_2H_4O_2H^+$	180	250
			$C_3H_6O_2H^+$	24	31
			$C_4H_8O_2H^+$	32	130
CxHy	170	260	$C_3H_6H^+$	14	17
			$C_4H_8H^+$	23	33
			$C_6H_{10}H^+$	17	23
N-containing	33	56	$C_2H_3NO_2H^+$	1.8	16
			$C_3H_7NOH^+$	3.8	7.9
CxHyO	580	680	$C_2H_2OH^+$	100	210
			Acetaldehyde*	60	29
			$C_6H_4OH^+$	9.5	62

* Acetaldehyde did not increase as the enthalpy increased

Table S5. Yield (ppb VOC per ppb ozone) of top ten species having the most increase under ozone-present condition for experiments of skin-only emissions with moderate and high relative humidity (RH).

**Exp. 13** Temperature: 30°C RH: 28%			**Exp.11** Temperature: 31°C RH: 70%		
Ranking	Species	Yield	Ranking	Species	Yield
1	6-MHO	0.105	1	6-MHO	0.119
2	4-OPA	0.118	2	4-OPA	0.139
3	Acetone	0.161	3	Acetone	0.193
4	Acetic acid	0.071	4	Acetic acid	0.171
5	$C_2H_2OH^+$	0.094	5	$C_2H_2OH^+$	0.194
6	$C_6H_{10}H^+$	0.041	6	$C_6H_{10}H^+$	0.065
7	Decanal	0.012	7	Decanal	0.029
8	Nonanal	0.012	8	Nonanal	0.025
9	1,4-butanedial	0.015	9	Nonenal	0.018
10	Nonenal	0.009	10	Acetaldehyde	0.027

Table S6. Human breath emission rates (μg h^{-1} p^{-1}) of selected species in the literature.

Species	Sun et al. (2017) Mean (range)	This study, Exp. 12* Mean over steady state
Acetone	24.84 (24.08 - 37.27)	760
Isoprene	36.71 (0.98 - 288.25)	250
Acetic acid	2.29 (0.16 – 11.8)	Not detected
Monoterpenes	6.51 (0.25 – 514.23)	2.4
Methyl ethyl ketone	0.44 (0.13 – 2.06)	Not detected
Phenol	4.8 (0.49 – 193.79)	Not detected

*Under ozone-free condition

Table S7. Human dermal emission rates (μg h^{-1} p^{-1}) of selected species in the literature.

Species	Zou et al. (2020) Mean of replicates for one subject	Mochalski et al. (2014) Mean of 10 subjects	This study, Exp. 13* Mean over steady state
Monoterpenes	2.6	6.2	7.3
Acetic acid	19.1	Not reported	190
$C_4H_8O_2$ (ester)	19.8	Not reported	32
Acetaldehyde	6.5	Not reported	60
Acetone	39.7	156	180
Methyl ethyl ketone	Not reported	17	7.9
Methyl vinyl ketone	Not reported	24.2	15
6-MHO	4	5	15
Geranyl acetone	8.8	Not reported	5.4
Decanal	39	Not reported	8.2
Nonanal	32.6	13	6.2
Octanal	5.4	7.6	5.5
Hexanal	6.5	11.9	1.2

*Under ozone-free condition

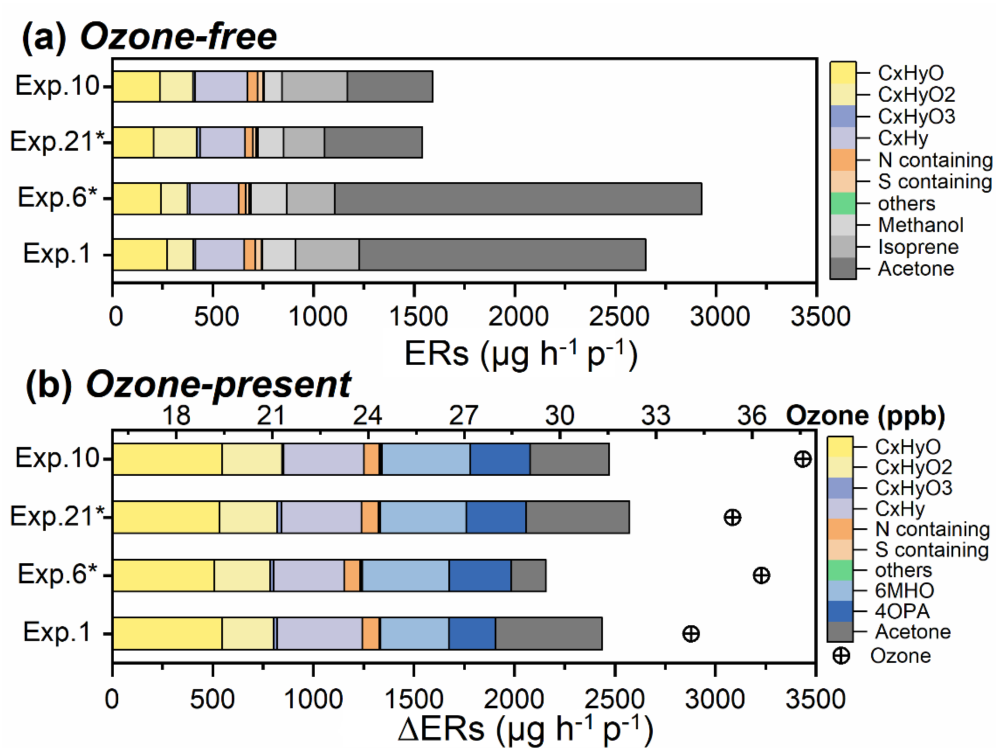

Figure S1. (a) human emission rates (ERs) under ozone-free condition and (b) the absolute change of human emission rates under ozone-present condition: ΔERs of top 3 contributing species and other species categorized into 7 subgroups for four benchmark experiments included in the study to derive the mean whole-body VOC emission rates. *Exp. 21 is the replicate of Exp. 6. Steady-state ozone concentration is shown in the lower panel (b).

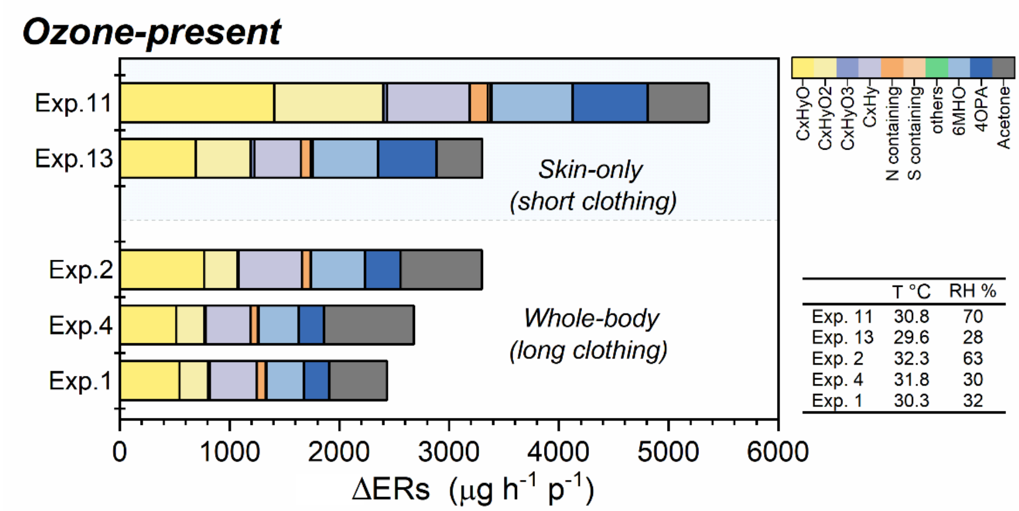

Figure S2. Fractional contributions to the absolute change of whole-body emission rates (ΔERs) for whole-body and skin-only emissions under different temperature and relative humidity under ozone-present condition.

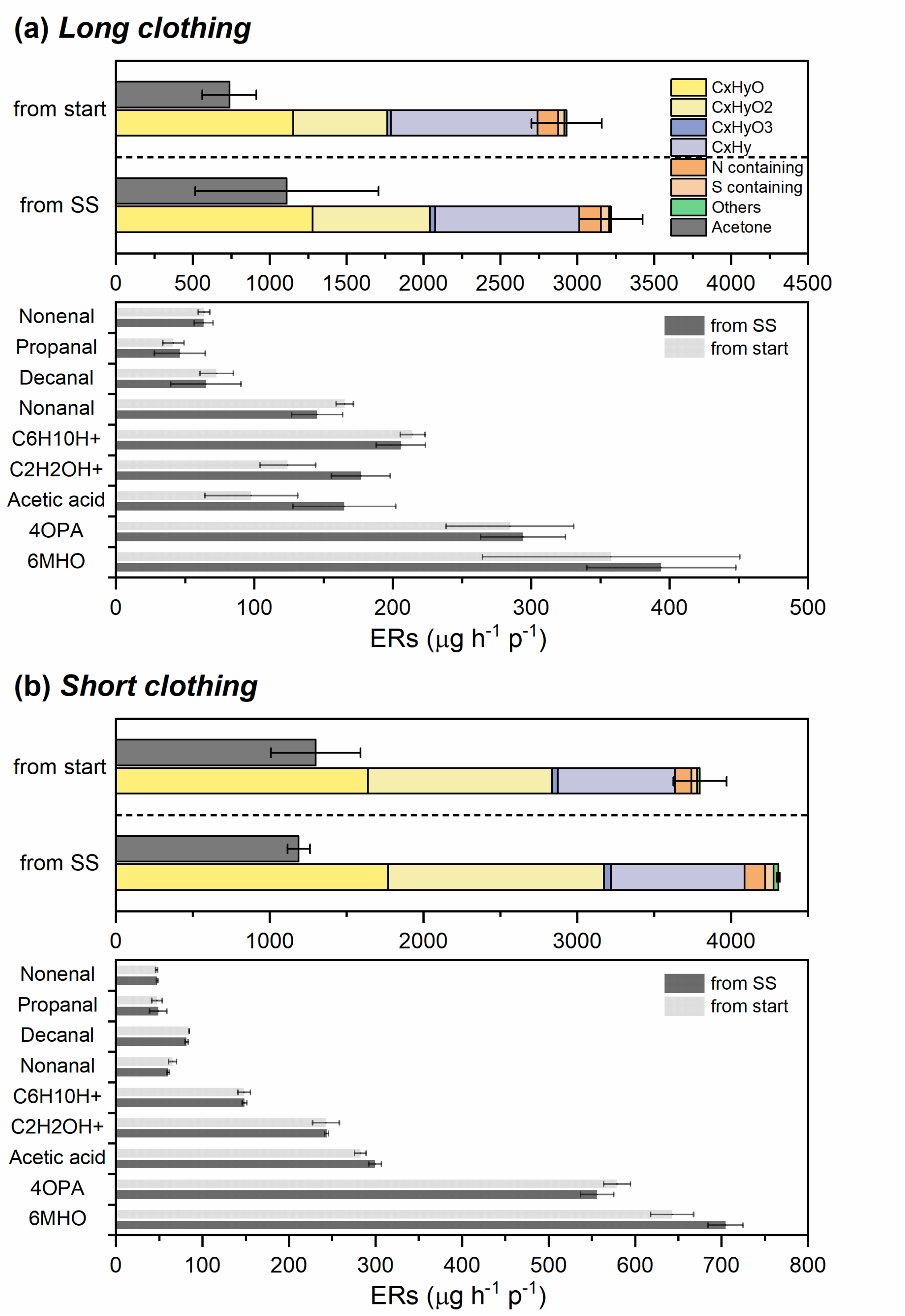

Figure S3. Fractional contributions to total ERs as well as the ERs of the top ten species (excluding acetone) for two types of experiments (from start: ozone dosing initiated before volunteers entered; from SS: ozone dosing initiated after human emissions reached steady-state), wearing (a) long clothing (from SS: N = 8; from start: N=4) and (b) short clothing (from SS: N = 2; from start: N=2). Error bars refer to the standard deviation among experiments included for each condition.

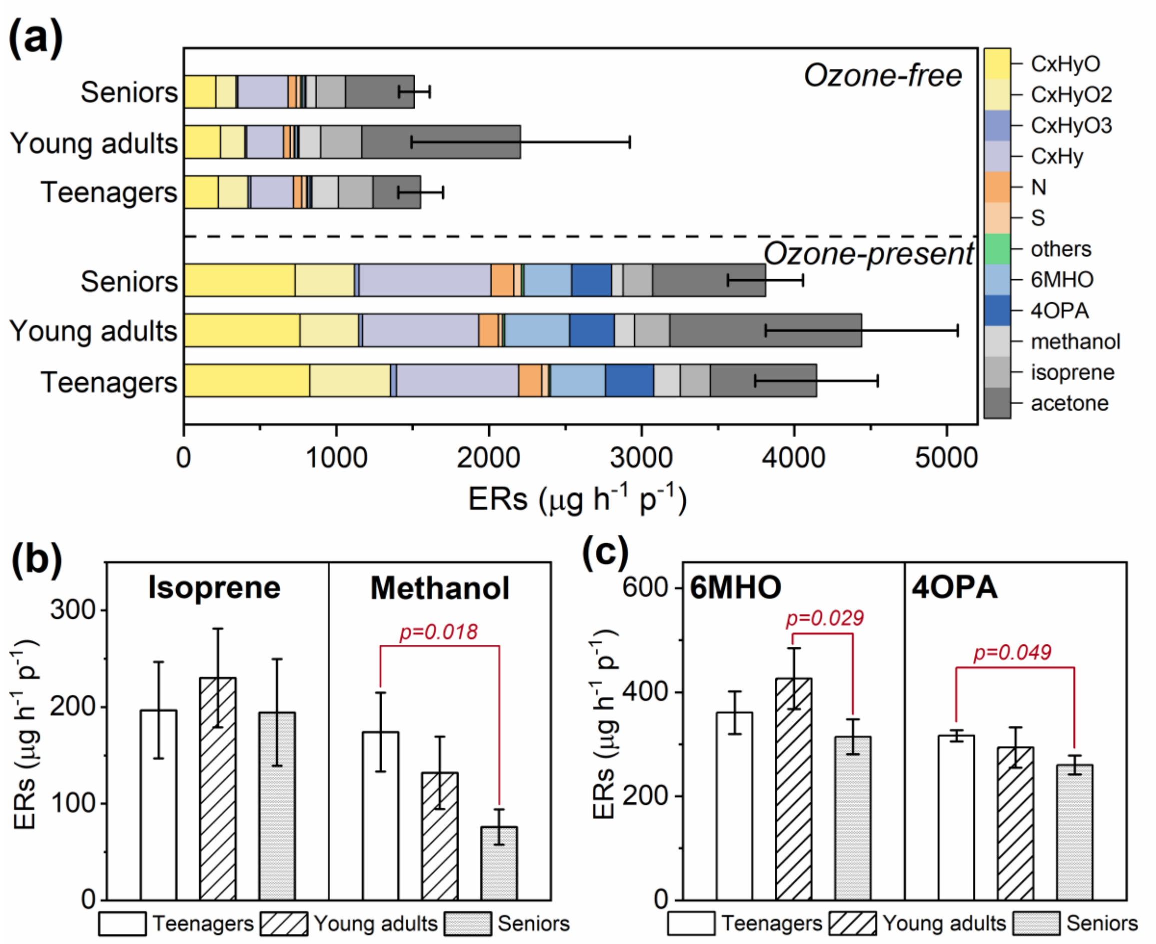

Figure S4. (a) fractional contributions to the human whole-body emission rates of teenagers, young adults and seniors under ozone-free and ozone-present conditions, (b) ERs of isoprene and methanol of different age groups under ozone-free condition, and (c) ERs of 6-MHO and 4-OPA of different age groups under ozone-present condition. Number of experiments included for ozone-free condition: 2 (teenagers), 2 (seniors), 4 (young adults); for ozone-present condition: 3 (teenagers), 3 (seniors), 6 (young adults).

References

Ager, C., Mochalski, P., King, J., Mayhew, C. A., and Unterkofler, K.: Effect of inhaled acetone concentrations on exhaled breath acetone concentrations at rest and during exercise, J Breath Res, 14, 026010, 2020.

Bekö, G., Wargocki, P., Wang, N., Li, M., Weschler, C. J., Morrison, G., Langer, S., Ernle, L., Licina, D., Yang, S., Zannoni, N., and Williams, J.: The Indoor Chemical Human Emissions and Reactivity (ICHEAR) project: Overview of experimental methodology and preliminary results, Indoor Air, 30, 1213-1228, 2020.

Cappellin, L., Karl, T., Probst, M., Ismailova, O., Winkler, P. M., Soukoulis, C., Aprea, E., Mark, T. D., Gasperi, F., and Biasioli, F.: On quantitative determination of volatile organic compound concentrations using proton transfer reaction time-of-flight mass spectrometry, Environ Sci Technol, 46, 2283-2290, 10.1021/es203985t, 2012.

Finlayson-Pitts, B. J., and Pitts Jr, J. N.: Chemistry of the upper and lower atmosphere: theory, experiments, and applications, Elsevier, 1999.

Holzinger, R.: PTRwid: A new widget tool for processing PTR-TOF-MS data, Atmospheric Measurement Techniques, 8, 3903-3922, 10.5194/amt-8-3903-2015, 2015.

IUPAC Task Group on Atmospheric Chemical Kinetic Data Evaluation: Datasheets - gas phase: http://iupac.pole-ether.fr/

Kramer, C., Mochalski, P., Unterkofler, K., Agapiou, A., Ruzsanyi, V., and Liedl, K. R.: Prediction of blood:air and fat:air partition coefficients of volatile organic compounds for the interpretation of data in breath gas analysis, Journal of Breath Research, 10, 017103, 2016.

Mochalski, P., Unterkofler, K., Hinterhuber, H., and Amann, A.: Monitoring of selected skin-borne volatile markers of entrapped humans by selective reagent ionization time of flight mass spectrometry in NO+ mode, Anal Chem, 86, 3915-3923, 2014.

Sekimoto, K., Li, S.-M., Yuan, B., Koss, A., Coggon, M., Warneke, C., and de Gouw, J.: Calculation of the sensitivity of proton-transfer-reaction mass spectrometry (PTR-MS) for organic trace gases using molecular properties, International Journal of Mass Spectrometry, 421, 71-94, 2017.

Smith, A. M., Rigler, E., Kwok, E. S. C., and Atkinson, R.: Kinetics and Products of the Gas-Phase Reactions of 6-Methyl-5-hepten-2-one and trans-Cinnamaldehyde with OH and NO3 Radicals and O3 at 296 ± 2 K, Environmental Science & Technology, 30, 1781-1785, 10.1021/es950871m, 1996.

Spanel, P., Dryahina, K., and Smith, D.: A quantitative study of the influence of inhaled compounds on their concentrations in exhaled breath, J Breath Res, 7, 017106, 2013.

Sun, X., He, J., and Yang, X.: Human breath as a source of VOCs in the built environment, Part II: Concentration levels, emission rates and factor analysis, Building and Environment, 123, 437-445, 2017.

Wang, N., Zannoni, N., Ernle, L., Bekö, G., Wargocki, P., Li, M., Weschler, C. J., and Williams, J.: Total OH Reactivity of Emissions from Humans: In Situ Measurement and Budget Analysis, Environmental Science & Technology, 55, 149-159, 10.1021/acs.est.0c04206, 2021.

Weschler, C. J., and Shields, H. C.: Production of the hydroxyl radical in indoor air, Environmental Science & Technology, 30, 3250-3258, 1996.

Wisthaler, A., and Weschler, C. J.: Reactions of ozone with human skin lipids: sources of carbonyls, dicarbonyls, and hydroxycarbonyls in indoor air, Proceedings of the National Academy of Sciences, 107, 6568-6575, 2010.

Zhao, J. and Zhang, R.: Proton transfer reaction rate constants between hydronium ion (H3O+) and volatile organic compounds, Atmospheric Environment, 38, 2177-2185, 2004.

Zhou, S., Gonzalez, L., Leithead, A., Finewax, Z., Thalman, R., Vlasenko, A., Vagle, S., Miller, L. A., Li, S. M., Bureekul, S., Furutani, H., Uematsu, M., Volkamer, R., and Abbatt, J.: Formation of gas-phase carbonyls from heterogeneous oxidation of polyunsaturated fatty acids at the air–water interface and of the sea surface microlayer, Atmospheric Chemistry and Physics, 14, 1371-1384, 10.5194/acp-14-1371-2014, 2014.

Zou, Z., He, J., and Yang, X.: An experimental method for measuring VOC emissions from individual human whole-body skin under controlled conditions, Building and Environment, 181, 107137, 2020.
